# Clinical study of isosorbide mononitrate treatment for angina pectoris in coronary heart disease

**DOI:** 10.3892/etm.2013.958

**Published:** 2013-02-18

**Authors:** ZHENG TAN, XIAOMING SHANG, LI LI, LUOBING TIAN, YI MA, YAN PENG, LINGXI GAO

**Affiliations:** 1 Department of Internal Medicine, Hebei Medical University, Shijiazhuang, Hebei 050017;; 2 Department of Cardiovasology, Tangshan Gongren Hospital, Hebei Medical University, Tangshan, Hebei 063000;; 3 Second Department of Cardiovasology, Tangshan Gongren Hospital, Hebei Medical University, Tangshan, Hebei 063000;; 4 Hebei United University, Tangshan, Hebei 063009, P.R. China

**Keywords:** isosorbide mononitrate, intravenous drip, angina pectoris, coronary heart disease

## Abstract

The aim of this study was to observe and investigate the clinical efficacy of an intravenous drip of isosorbide mononitrate (ISMN) for the treatment of angina pectoris in coronary heart disease. A total of 102 patients with angina pectoris in coronary heart disease were divided into two groups. For the treatment group (n=51), 20 mg ISMN was added to 250 ml 0.9% normal saline and administered by intravenous drip for 14 consecutive days, twice daily. For the control group (n=51), 10 mg glyceryl trinitrate was added to 250 ml 0.9% normal saline and administered by intravenous drip for 14 consecutive days, twice daily. The clinical efficacy and adverse reactions were compared between the two groups. The disease symptoms of the two groups were improved. Compared with the control group, the clinical efficacy and electrocardiogram examination results of the treatment group were significantly improved (P<0.05). The intravenous formulation of ISMN is an effective treatment for angina pectoris in coronary heart disease and it has the advantages of fewer adverse reactions and higher safety.

## Introduction

With the continuously increasing aging of the population and changes to influencing factors, including lifestyle, disease spectra have significantly changed. Cardiovascular disease has become the main disease harming human health and quality of life and its morbidity rate presents a rising trend. Additionally, its disability and mortality rates have increased. According to the World Health Organization (WHO) statistics in 2011, 17.10 million individuals succumb to cardiovascular disease each year worldwide, accounting for 29% of the total mortalities due to various causes. It is estimated that this figure will rise to 25 million by 2020. Of these, 80% patients are from developing countries.

As the standards of living and lifestyle are continuously changing, the morbidity and mortality rates of coronary heart disease in China also present a rising trend. Therefore, it is important to further investigate this disease. In coronary heart disease, there are a number of pathogenic factors, including stress (1), depression (2), bad habits (alcohol abuse, excessive smoking, staying up late, lack of exercise, etc.) and air pollution (3–5). Coronary heart disease is a common clinical disease, caused by vascular stenosis or obstruction due to coronary atherosclerosis or myocardial ischemia, or necrosis due to functional changes of the coronary arteries (convulsion), also called ischemic heart disease. Due to its high morbidity and mortality rates, it is considered the primary disease affecting human health and quality of life (6,7).

Angina pectoris is a clinical syndrome caused by myocardial sharp temporary ischemia and hypoxia due to insufficient coronary blood supply (8). Its common causes include over-straining, agitation and poor dietary habits (9). Moreover, it is more common in females than in males (10,11). As age increases, the morbidity rate of angina pectoris gradually rises. Following the onset of angina pectoris, ∼5% patients are likely to succumb to coronary heart disease within 5 years. The incidence rate of acute myocardial infarction in males with angina pectoris in coronary heart disease is higher compared with that in females (12).

In the clinic, there are a number of drugs and methods that are used for treating this disease, and nitrate ester drugs are the preferred treatment. Among them, isosorbide mononitrate (ISMN) is a long-acting nitrate ester drug and is the main active metabolite of isosorbide dinitrate. ISMN not only dilates the arteries and veins to reduce the cardiac preload and postload (13), but also dilates normal or constrictive coronary arteries to improve myocardial ischemia and ventricular systolic function (14). Although there are relevant reports on the ISMN treatment of coronary heart disease in the clinic, the sample size is often small and case information is often incomplete. For a more comprehensive and specific study of the efficacy of an intravenous drip of ISMN in the treatment of angina pectoris in coronary heart disease, we used ISMN to treat 102 patients with angina pectoris in coronary heart disease from December 2010 to June 2012.

## Patients and methods

### General data

According to the diagnostic criteria of angina pectoris in coronary heart disease prepared by WHO in 1979 (6), 102 patients with angina pectoris in coronary heart disease who were hospitalized in the cardiovascular wards of Tangshan Gongren Hospital from December 2010 to June 2012 were selected and randomly divided into two groups (Table I). Among them, there were 51 cases in the treatment group, including 23 males and 28 females. Their mean age was 47.6±7.0 years and the disease course of coronary heart disease was 5.3±1.6 years. In the control group, there were 51 cases, including 24 males and 27 females. Their mean age was 46.5±8.6 years and the disease course of coronary heart disease was 5.6±1.2 years. There was no significant difference in gender, age, hypertension, smoking, diabetes mellitus, hypercholesterolemia and angina pectoris history between the two groups of patients (P<0.05). This study was conducted in accordance with the Declaration of Helsinki and with approval from the Ethics Committee of Hebei Medical University. Written informed consent was obtained from all participants. At 24 h before administration, drugs affecting myocardial blood supply were stopped.

### Administration method

For the control group, 10 mg glyceryl trinitrate injection was added to 250 ml 0.9% normal saline and was administered by intravenous drip. For the treatment group, 20 mg ISMN injection (Xinkang Injection; Lunan Pharmaceutical Co., Ltd., Linyi, China; batch number, 01010121) was added to 250 ml 0.9% normal saline by intravenous drip (glucose injection was used for patients with glucopenia). For the two groups, the drip rate was maintained at 2 ml/min. Administration was conducted twice daily and the treatment course was 14 days. During treatment, buccal glyceryl trinitrate was administered in case of angina pectoris attack. In addition, the patients with complicated primary hypertension and diabetes mellitus were treated accordingly. Moreover, electrocardiogram and liver and kidney function examinations were conducted regularly to observe changes of angina pectoris attack frequency in the two groups prior to and following treatment. The electrocardiogram ST stage and T wave were also compared prior to and following treatment. Additionally, side reactions were noted.

### Observed parameters

For all patients, venous blood was drawn prior to, during and following treatment. Additionally, liver and kidney function, blood rheology-related indicators, blood routine, urine routine and electrocardiogram examinations were conducted. During medication, angina pectoris attack frequency, attack extent at the attack site, as well as duration and glyceryl trinitrate dosage were observed and recorded daily. At the same time, changes in the main symptoms prior to and following treatment, as well as adverse reactions were recorded.

### Evaluation criteria of clinical efficacy

According to the clinical manifestations of patients, clinical efficacy was divided into extremely effective, effective and ineffective. For the extremely effective level, the angina pectoris symptoms disappeared and the fatigue extent of angina pectoris attack frequency and ISMN consumption amount reduced by >80%. For the effective level, the angina pectoris symptoms were clearly improved and the fatigue extent of angina pectoris attack frequency and ISMN consumption amount reduced by 50–80%. For the ineffective level, the angina pectoris symptoms were not relieved and attack frequency and ISMN consumption amount reduced by <50%.

### Electrocardiographic criteria for evaluation of efficacy

According to the improvement of the electrocardiogram ST-T wave, the clinical efficacy was divided into extremely effective, effective and ineffective. For the extremely effective level, ST-T changes of R wave in lead on the electrocardiogram recovered to the normal level, or exercise tolerance rose by two grades and the exercise test electrocardiogram changed from positive to negative. For the effective level, ST-T changes of R wave in lead on the electrocardiogram dropped by >0.1 mV, T wave inversion became shallower by >50%, the T wave changed from evenness to erection, or exercise tolerance rose by one grade. For the ineffective level, the resting electrocardiogram did not change.

### Evaluation of adverse reactions

Adverse reactions included giddiness, headache, nausea, vomiting, flushed face, blood routine abnormalities, palpitation, gastrointestinal tract hemorrhage, asthmatic attack, anaphylactic reaction and rhinitis following administration. The attack time, severity extent, duration, test measurements and sequelae were jointly recorded by observation physicians and nursing staff. Severe adverse reactions, including hematencephalon, gastrointestinal hemorrhage disability and mortality were reported to the Ethics Committee for National Clinical Research Base, the drug manufacturer and the Drug Research Supervision Office, Safety Supervision Department of State Food and Drug Administration.

### Statistical analysis

SPSS 20 software (SPSS Inc., Chicago, IL, USA) was used for statistical analysis. The significance level was set at α=0.05. A t-test was used for comparison of measurement data and χ^2^ test was used for enumeration data.

## Results

### Comparison of clinical efficacy between the two groups

In the 51 cases in the treatment group, the clinical efficacy of glyceryl trinitrate in 26 cases was extremely effective, 22 cases was effective and 3 cases was not effective. The total effective rate was 94.1%. Among the 51 cases in the control group, the clinical efficacy of ISMN in 15 cases was extremely effective, 21 cases was effective and 15 cases was not effective. The total effective rate was 70.6%. A significant difference (P<0.05) was observed between the two groups (Table II).

### Comparison of electrocardiogram results between the two groups

Compared with prior to administration in the treatment group, the clinical efficacy in 19 cases was extremely effective, 26 cases was effective and 6 cases was not effective. Among the 51 cases in the control group, the clinical efficacy in 9 cases was extremely effective, 24 cases was effective and 10 cases was not effective. The total effective rate of the treatment group (88.2%) was significantly higher compared with that of the control group (64.7%; Table III).

### Comparison of adverse reactions between the two groups

Among the 51 cases in the treatment group, 3 cases presented flushed face, giddiness and other symptoms during treatment. Among the 51 cases in the control group, 6 cases presented giddiness, palpitation, headache, nausea and other symptoms during treatment and the symptoms were relieved after slowing the drip rate. After several days, the symptoms disappeared, which did not affect further treatment. For the two groups of patients, there was no significant change in blood and urine routine test results, as well as liver and kidney function, blood lipid, blood glucose and electrolyte results prior to and following treatment.

## Discussion

Coronary heart disease, also known as ischemic heart disease, is caused by vascular stenosis and obstruction, as well as blood flow obstruction due to coronary atherosclerosis. The balance of blood and oxygen supply with myocardial need is affected by damage to the coronary arteries; therefore, coronary blood flow does not meet myocardial need, which causes myocardial acute ischemia and hypoxia, resulting in angina pectoris. Angina pectoris is one of the most common types of coronary heart disease and its clinical manifestation is a paroxysmal crushing pain generated at the pleural center (15), which occasionally migrates to the neck, jaw, arm, back and stomach. The morbidity rate of coronary heart disease presents a rising trend and is higher in younger individuals. Coronary heart disease is a serious threat to human life. The main pathogenic factors include smoking, hypertension, hyperlipidemia and diabetes mellitus. Studies have demonstrated that high-sensitivity C-reactive protein (hs-CPR) is an independent risk factor of coronary heart disease and is closely related to the occurrence and development of the disease (16–19). The key for treating angina pectoris in coronary heart disease is to control or slow down arteriosclerosis, reduce blood lipid levels and blood viscosity, reduce platelet aggregation, protect vascular endothelial function, dilate coronary arteries and improve myocardial oxygen and oxygen supply (20–22). ISMN is the main metabolite of isosorbide dinitrate and is the preferred drug for treating angina pectoris in coronary heart disease in the clinic (20).

In this study, 102 patients with angina pectoris in coronary heart disease were selected. A detailed, specific and comprehensive exploratory study was conducted to obtain complete medical information and follow-up data to determine the efficacy of an intravenous drip of ISMN in treating angina pectoris in coronary heart disease. We identified that an intravenous drip of ISMN is effective in treating angina pectoris in coronary heart disease. Intravenous ISMN for the treatment of angina pectoris is effective since the drug is able to rapidly distribute to the whole body. Additionally, it is hardly metabolized in the liver and has no hepatic or intestinal side-effects. It has a complete and absolute bioavailability (23), its half-life is 4–5 h and its effective blood rheology effect is ∼8 h, 10-fold more than that of glyceryl trinitrate (24). Its main pharmacological effects are relaxation of smooth muscle, dilatation of coronary arteries and veins, reduction of left ventricular end diastolic pressure and pulmonary capillary wedge pressure (preload), improvement of myocardial blood supply, reduction of myocardial oxygen consumption and reduction of systolic arterial pressure and mean arterial pressure (postload). In addition, it provides exogenous nitric oxide, platelet adhesion and aggregation, and vascular smooth muscle cell proliferation. The above effects jointly cause myocardial oxygen consumption to decrease and oxygen supply to increase and thus relieves angina pectoris. According to the above analyses, ISMN is a strong, long-acting drug for the short- and long-term treatment of angina pectoris. However, there are a number of problems and defects in this study due to time restraints, research conditions and experience. The current clinical observation indicators and efficacy evaluation measures were more simple and clear, and the efficacy evaluation was mainly conducted according to clinical symptoms and electrocardiogram examination. It is necessary to add more specific efficacy evaluation indicators, including coronary artery angiography. Moreover, the study methods are under-developed and the controlled observations of a randomized blind method were not applied. Therefore, the objective efficacy of ISMN in treating coronary heart disease may not be adequately embodied. In addition, the long-term follow-up for patients was not sufficient; therefore, it is necessary to conduct a clinical study under prospective randomized, double-blind research conditions with long-term follow-up, in order to confirm the clinical efficacy of ISMN in the treatment of angina pectoris in coronary heart disease.

According to the clinical observations in this study, the intravenous drip of ISMN is fast-acting with good absorption, high bioavailability, clear efficacy and high safety in the treatment of angina pectoris in coronary heart disease. Compared with glyceryl trinitrate, it has characteristics of long action duration and few side-effects and is worthy of promotion and application in the clinic.

## Figures and Tables

**Table I:** Comparison of case information between the two groups ().

Group	N (male/female)	Age (years^a^)	Disease course (years^a^)
Treatment	51 (23/28)	47.6±7.0	5.3±1.6
Control	51 (24/27)	46.5±8.6	5.6±1.2

a mean ± SD.

**Table II:** Comparison of clinical efficacy between the two groups (P<0.005).

Group	N	Extremely effective n (%)	Effective n (%)	Ineffective n (%)	Total effective rate (%)
Treatment	51	26 (50.9)	22 (43.1)	3 (5.9)	94.1
Control	51	15 (30.0)	21 (41.2)	15 (29.4)	70.6

**Table III:** Comparison of efficacy between the two groups by electrocardiography (P<0.05).

Group	N	Extremely effective n (%)	Effective n (%)	Ineffective n (%)	Total effective rate (%)
Treatment	51	19 (37.3)	26 (51.0)	6 (11.8)	88.2
Control	51	9 (17.6)	24 (47.1)	10 (19.6)	64.7
